# Microbial Community Diversities and Taxa Abundances in Soils along a Seven-Year Gradient of Potato Monoculture Using High Throughput Pyrosequencing Approach

**DOI:** 10.1371/journal.pone.0086610

**Published:** 2014-01-30

**Authors:** Xing Liu, Junlian Zhang, Tianyu Gu, Wenming Zhang, Qirong Shen, Shixue Yin, Huizhen Qiu

**Affiliations:** 1 College of Resources and Environmental Sciences, Gansu Agricultural University, Lanzhou, Gansu Province, China; 2 Gansu Provincial Key Laboratory of Aridland Crop Science, Gansu Agricultural University, Lanzhou, Gansu Province, China; 3 College of Agronomy, Gansu Agricultural University, Lanzhou, Gansu Province, China; 4 Gansu Key Laboratory of Crop Genetic and Germplasm Enhancement, Gansu Agricultural University, Lanzhou, China; 5 College of Environmental Science and Engineering, Yangzhou University, Yangzhou, Jaingsu Province, China; 6 Jiangsu Provincial Key Lab of Organic Solid Waste Utilization, Nanjing Agricultural University, Nanjing, China; University of Missouri, United States of America

## Abstract

**Background:**

Previous studies have focused on linking soil community structure, diversity, or specific taxa to disturbances. Relatively little attention has been directed to crop monoculture soils, particularly potato monoculture. Information about microbial community changes over time between monoculture and non-monoculture treatments is lacking. Furthermore, few studies have examined microbial communities in potato monoculture soils using a high throughput pyrosequencing approach.

**Methodology/Principal Findings:**

Soils along a seven-year gradient of potato monoculture were collected and microbial communities were characterized using high throughput pyrosequencing approach. Principal findings are as follows. First, diversity (*H*_Shannon_) and richness (*S*_Chao1_) indices of bacterial community, but not of fungal community, were linearly decreased over time and corresponded to a decline of soil sustainability represented by yield decline and disease incidence increase. Second, *Fusarium*, the only soilborne pathogen-associated fungal genus substantially detected, was linearly increased over time in abundance and was closely associated with yield decline. Third, *Fusarium* abundance was negatively correlated with soil organic matter (OM) and total nitrogen (TN) but positively with electrical conductivity (EC). Fourth, *Fusarium* was correlated in abundances with 6 bacterial taxa over time.

**Conclusions:**

Soil bacterial and fungal communities exhibited differential responses to the potato monoculture. The overall soil bacterial communities were shaped by potato monoculture. *Fusarium* was the only soilborne pathogen-associated genus associated with disease incidence increase and yield decline. The changes of soil OM, TN and EC were responsible for *Fusarium* enrichment, in addition to selections by the monoculture crop. *Acidobacteria* and *Nitrospirae* were linearly decreased over time in abundance, corresponding to the decrease of OM, suggesting their similar ecophysiologial trait. Correlations between abundance of *Fusarium* with several other bacterial taxa suggested their similar behaviors in responses to potato monoculture and/or soil variables, providing insights into the ecological behaviors of these taxa in the environment.

## Introduction

Soil microbial community is extremely important in maintaining soil quality, functions, sustainability and global nutrient cycling. Majority of past studies focused on linking the microbial community structure, diversity, or specific taxa to soil variables/disturbances, such as soil pH [1], [2], soil types [3], soil texture [4], soil layers [5], plant species [6], nitrogen level [7], temperature [8], water regime [9], tillage [10], [11], amendments [12], biocontrol agents [13], land uses [14], and contaminations [15]. However, relatively limited attention has been directed to the links between crop monoculture and soil microbial communities.

Crop monoculture is when the same crop is grown year after year on the same soil, without interruption crops that could potentially induce different crop-specific microbial communities [16]. Crop monoculture can be viewed as a simple and an ideal model system to study the relationship between agricultural practices and soil microbial communities. When crop monoculture is practiced, the microbial community is continuously exposed to the roots of the same crop that selects and enriches certain groups of microorganisms including yield-debilitating populations (i. e. soilborne pathogens) of that crop [17]. This consequently shapes the soil microbial community that can often be easily observed. Several studies have determined differences of soil microbial communities between rotation/mixed culture and monoculture of cotton [18], maize [19], wheat (and maize) [20], rice [21], soybean [22], oilseed rape [23] and potato [24]. With a few notable exceptions [24], these studies typically used an one time-point soil sampling strategy. Soil samples were collected at a given time-point after a certain time span, generally years, over which the monoculture was practiced. The microbial communities were then compared between monoculture and non-monoculture treatments. This sampling strategy is useful for revealing differences in communities between treatments, but it does not allow measurements of community changes over time (i. e. the number of years a crop monoculture has been maintained). This is important information to understand the assembly processes of microbial communities under continuous selection of crop monoculture practice [25], [26], [27], [28].

An alternative strategy is to sample soils along a year-gradient of monoculture. This strategy allows observations of changes in patterns of different microbial taxa over the years that may occur in a predictable manner within the experimental time span. In addition, the change pattern of one taxon may be correlated with another in abundance, allowing us to perceive the behaviors of these taxa in response to potato monoculture. In spite of these benefits, studies using year-gradient samples are limited in literature. Some early reports followed this strategy (see review [29]), but community changes were based on culture-dependent methods only, which have been proven to have strong biases against yet-to-cultured majorities [30]. The relationship between crop monoculture practices and the soil microbial community is thus not sufficiently understood. Relevant knowledge on potato monoculture agroecosystems is particularly lacking.

In a previous study [31], we examined the fungal community in potato monoculture soils. Further re-examination of our original data revealed that the diversity (*H*_Shannon_) and richness (*S*_Chao1_) indices of the fungal community were nearly un-changed over the years of monoculture (time). Furthermore, *Fusarium* was the only soilborne pathogen-associated fungal genus substantially detected using a 454 pyrosequencing approach. *Fusarium* was significantly enriched over time, and closely associated with disease incidence observed in the field (see the Results section below). These findings encouraged us to further examine whether the diversity and richness indices of the bacterial community were also significantly changed. Specifically we are interested in knowing change patterns of different bacterial taxa with reference to that of *Fusarium*. Based on the above findings, we hypothesized that the overall soil bacterial community were significantly changed under the continuous monoculture pressure, and abundance of some bacterial taxa were correlated with *Fusarium* abundances over time. As soil variables play key roles in microbial community structuring[1], [7], [14], associations between soil variables and the microbial change patterns were also studied.

## Materials and Methods

### Ethics Statement

Authors confirm that no specific permissions were required for the locations/activities involved in this study, because the land accessed is publically owned and no protected species were sampled. Authors also confirm that the field studies did not involve endangered or protected species.

### Field experiment, soil sampling and variable measurements

Detailed descriptions of the field experiment, soil sampling and variable measurements can be found elsewhere [31]. Only a brief description is given here. The experimental field, located at the Tiaoshan Farm, a warm terrestrial arid area with a mean annual precipitation 186 mm is located in Gansu Province, China. This field contained 21 plots, each being 9.0×6.1 m in size. Prior to potato monoculture, the field was under a corn-potato rotation. Each year, starting from year 2005, 3 plots (replicates) were selected for practicing potato (“Atlantic” cultivar) monoculture, leaving other plots to continue with the corn-potato rotation. The previous crop of selected plots or the potato year of the rotation was always corn. By 2011, all plots were used up. This experimental design provides opportunity to collect soil samples after potato monoculture from 1 to 7 years simultaneously.

Potato was typically seeded in April 25 with a few days variation among years. Tuber pieces were buried on the top of raised paths (∼40 cm in height and ∼135 cm in bottom width) and plants were spaced 17 cm apart along the row. Two rows were planted on each raised path with 70 cm between the two rows. This resulted in a plant density of 460 plants per plot (equivalent to 84,075 plants ha^−1^). Blended fertilizer (15-15-15), additionally supplemented with urea and K_2_SO_4_, was used at the rate of 210 kg N ha^−1^, with the ratio N:P_2_O_5_:K_2_O being 1.4∶1.0∶2.0. Nitrogen form in blended fertilizer was (NH_4_)_2_SO_4_. Fertilizers were applied before seeding by machine. All other field management activities were performed manually. Once seeded and fertilized, the raised paths were covered manually with a plastic film. The field was irrigated by introducing water into the ditch between two raised paths through pipes. Irrigation was done three times during the growth period, typically at June 1 (seedling stage), July 1 (early flowering stage) and July 20 (tuber enlargement stage) at the rate of 2,700 t ha^−1^ each time. Potato was harvested in later August. For yield evaluation, the tubers were dug out manually. Stolons, underground stems, true roots, scale leaf rhizomes and adhered soils were removed. Tubers smaller than 2 cm in diameter were considered as developing tubers and were excluded from the yield calculations. Disease incidence was calculated by totaling the number of plants with symptoms of any recognizable disease, and converting to percent by dividing by the total number of plants and then multiplying by 100. Fusarium wilt was the major disease, covering ∼90% of diseased plants. The disease incidence was recorded three times during the growth period, but only the records at harvest time are presented.

Soil samples were collected in July 20, 2011. The plots with 5 years of monoculture were not sampled. From each plot, 15 spots were randomly sampled from the 0–20 cm soil layer and the soil from these spots were placed into a single aggregate sample and then well mixed. Totally, 18 samples were obtained. Samples were put into sterile plastic bags, placed into an ice box, transported to laboratory and used as soon as possible, or stored in a refrigerator at −80°C if not immediately used. Selected soil variables measured included organic matter (OM, by dichromate oxidation), total nitrogen (TN, by total Kjeldahl N), NH_4_^+^–N, NO_3_^−^–N (by 1 M KCl extraction), pH and electrical conductivity (EC) (both at 5∶1, water:soil ratio).

### DNA extraction, amplification, sequencing and sequence treatment

For each soil sample, bulk DNA was extracted from three 0.5 g aliquots of soils (dry weight basis) with a FastDNA SPIN Kit for Soil (Bio 101, Carlsbad, Calif.) following the manufacturer's instructions. The triplicate DNA samples were pooled together, resulting 18 DNA samples in total, representing 18 field plots. The integrity of the extracted DNA was confirmed by running on 0.8% agarose gel with 0.5 TBE buffer (45 mM Tris-borate, 1 mM EDTA, pH 8.0). The extracted DNA (as well as PCR products) was quantified using TBS-380 Mini-Fluorometer (Turner Biosystems, CA, USA). All DNA samples were diluted to 100 ng µl^−1^ and used as PCR templates.

For the fungal community, detail description on primer pairs, amplification, sequencing and sequence treatment can be found elsewhere [31]. Operational taxonomic units (OTUs) were assigned at the 97% identity level.

For the bacterial community, the primer pair 563F (5′-AYTGGGYDTAAAGVG-3′) and 802R (5′-TACNVGGGTATCTAATCC-3′) [32] was used for amplifying the V4 hypervariable region of the 16 S rRNA gene. The PCR mixture (20 µl) contained 5×FastPfu buffer 4 µl, 2.5 mM dNTPs 2 µl, each primer(5 µM) 0.4 µl, DNA 0.5 µl, FastPfu polymerase 0.4 µl, and ddH_2_O (TransGen Biotech, Beijing, China). DNA samples were amplified using the following program: 95°C 2 min, 30 cycles: 95°C 30 sec, 55°C 30 sec, 72°C 30 sec; 72°C 5 min; 10°C forever. A second round of PCR was performed under the same conditions using Amplicon Fusion Primers 5′-*A*-*x*-563F-3′ and 5′-*B*-802R-3′, where *A* and *B* represent the pyrosequencing adaptors (CCATCTCATCCCTGCGTGTCTCCGACGACT and CCTATCCCCTGTGTGCCTTGGCAGTCGACT) and *x* represents an 10 bp-tag for sample identification. For each sample, three independent PCRs were performed. The triplicate products were pooled and purified using AxyPrep PCR Clean-up Kit (Axygen Biosciences, CA, USA). The purified PCR products from different soil samples contained 1.06∼3.30 ng µl^−1^ DNA and were pooled in equal quantities for pyrosequencing in one run, which was performed commercially at Shanghai Majorbio Bio-pharm Biotechnology Co., Ltd., on a Roche GS 454 FLX platform (http://www.majorbio.com).

Sequence reads were treated using MOTHUR v. 1.29.2 [33]. The forward primer (563F), tags and adaptor *A* and *B* were removed. Low quality nucleotides were filtered using qwindowaverage 27 and qwindowsize 50. Reads shorter than 120 bp were removed. Filtered sequences were aligned against Silva database (106 v.). Chimera reads were removed using both *chimera.slayer* and *chimera.uchime* scripts. A few reads belonging to *Archaea* were excluded. OTUs were assigned at the 97% identity level. On average, 11,950 original reads per sample were obtained, and after filtering 8,750 high-quality sequences per sample were left, of which 97.5% were 155 bp in length. The OTU counts were normalized into relative abundances (%).

Statistics was done with SPSS (v 19) and curve fitting with Sigmaplot (v. 12.5). Unless stated, *p* values <0.05 were considered significant. Raw sequence data for bacteria and fungi were submitted to DDBJ database under the accession number DRA001116 and DRA000962 respectively.

## Results

### Overall microbial communities

**Figure 1:**
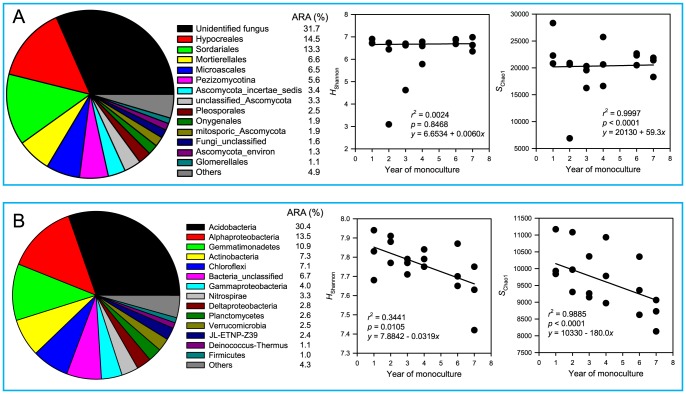
Overall fungal (A) and bacterial (B) communities in the soil samples along a year-gradient of potato monoculture. Pie charts show the average relative abundance over 18 samples. OTUs were assigned at the 97% identity level, based on which the *H*_Shannon_ and *S*_Chao1_ indices were calculated, which are shown by graphs. Phylogenetic lineages of bacteria were assigned by phylum/subphylum and of fungi by order or higher levels. ARA stands for average relative abundance. The “unidentified fungus” is a taxon able to be assigned to kingdom Fungi but unable to any lower taxon.

The overall bacterial communities are summarized in Figure 1. For comparison purposes, the summarized fungal community is also shown. Average relative abundances are listed in a column to the right of the pie chart. As for bacteria, *Acidobacteria*, α-*Proteobacteria* and *Gemmatimonadetes* were the top three abundant phyla with average relative abundances over 10%. As for fungi, “unidentified fungus”, *Hypocreales* and *Sordariales* were the top three abundant taxa. All the fungal taxa in Figure 1A, except “unidentified fungus” and some “others”, belong to phylum *Ascomycota*, which accounted for 58.2% in average relative abundance. *Basidiomycota* accounted for only 0.6%. The richness (*S*_Chao1_) and diversity (*H*_Shannon_) indices of the fungal community showed nearly flat lines over time, with slightly increasing trends by fitting to a linear model. Note that the *p* value of the fitted line for the fungal diversity index was not significant. In contrast, the richness and diversity indices of the bacterial community showed significant decreasing trends over time.

The change trends in relative abundances of these fungal taxa over 18 samples were shown in Figure S1. By curve fitting, the abundances of *Hypocreales, Pezizomycotina, Onygenales* and “fungi unclassified” showed significant increasing trends over time whereas *Mortierellales* and “*Ascomycota* incertae sedis” showed decreasing trends. By *p* values, *Hypocreales* was best fitted. *Hypocreales* and *Sordariales* were further broken down into genera and their relative abundances across soil samples were listed in Table S1. Within order *Hypocreales*, *Fusarium* was the top abundant genus with an average relative abundance 8%. All other genera were 1% or below in abundance. Within order *Sordariales*, *Chaetomium* and *Thielavia* were the top abundant genera with average relative abundances over 3%. All other genera in this order (except “unidentified fungus”) were 1% or lower in abundance.

### Yield trend and *Fusarium* abundance

**Figure 2:**
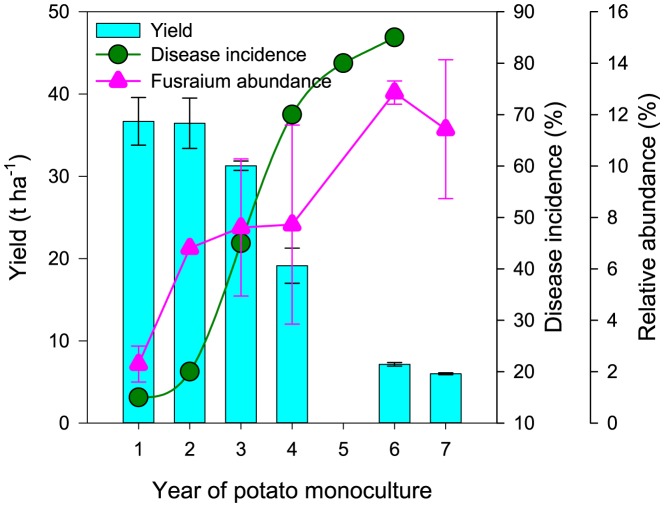
The change of potato yield, disease incidence and *Fusarium* abundance over the years of potato monoculture. Disease incidences shown in this figure were measured at harvest time.

Potato yield declined substantially after 2 years of monoculture, and was accompanied by an increase in disease incidence (Figure 2). Major diseases found in the field were fungi-associated, such as fusarium dry rots, late bright and black scurf/stem canker [31] that are caused by *Fusarium* spp., *Phytophthora infestans*, and *Rhizoctonia solani*, respectively. Because all these pathogens are soilborne, we further looked at how abundant they were in the soils. Because phytopathogens are often species-, even variety-specific, a pathogen in a strict taxonomic sense must be identified by pure culture, and can not be identified by environmental DNA-based approaches only (as in this present study). Therefore, we grouped the fungal abundances by genus that were detected in the soil samples, and checked them from a list of well-recognized potential soilborne potato pathogens [34], [35] by genus. If found among the list, the genera detected were referred to as pathogen-associated genera. Obviously we are not saying all *Fusarium* are pathogens, but viewing the data in this way showed that *Fusarium* was the only pathogen-associated fungal genus substantially detected in our soil samples with a range of relative abundance from 1.9% to 14.1% (Table S2). Other pathogen-associated genera were either below the detection limit (e. g. *Phytophthora* and *Rhizoctonia*) or detected but with too low relative abundances (e. g. *Phoma* and *Pythium*). The relative abundance of *Fusarium* increased with the increase of monoculture time, with the increase of disease incidence, and with the decrease of yield (Figure 2).

### Correlation between *Fusarium* abundance and soil variables

**Figure 3:**
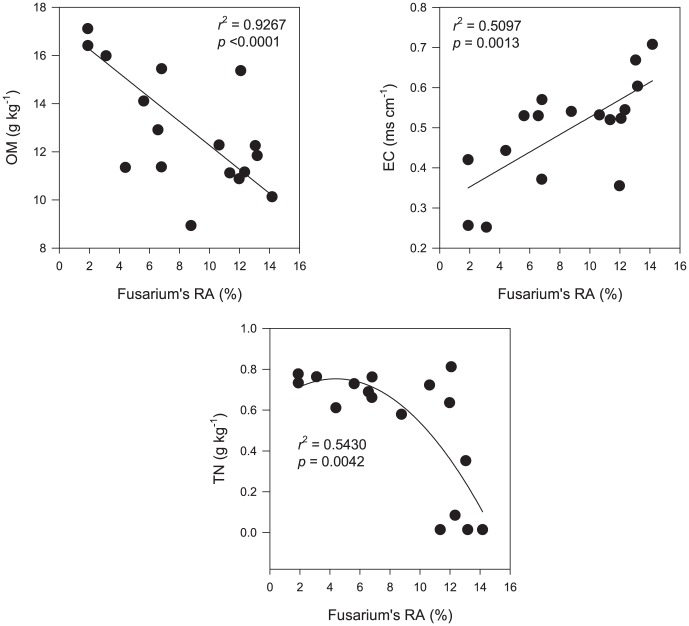
The correlations between *Fusarium* abundances and soil variables.

Using a linear model to fit soil variables with the year of monoculture, soil EC and pH were significantly increased over time, whereas OM, TN, and NH_4_^+^-N were decreased (Figure S2). NO_3_^−^-N was also decreased but did not fit the model. Further fitting soil variables with *Fusarium* abundance, it was shown that only OM, TN and EC were significantly correlated with the relative abundance of *Fusarium* (Figure 3). Other soil variables (NH_4_^+^-N and pH) were not significantly correlated, or not fitted with any of the models integrated in the software (NO_3_^−^-N).

### Correlation between abundance of bacterial taxa and the year of monoculture

**Figure 4:**
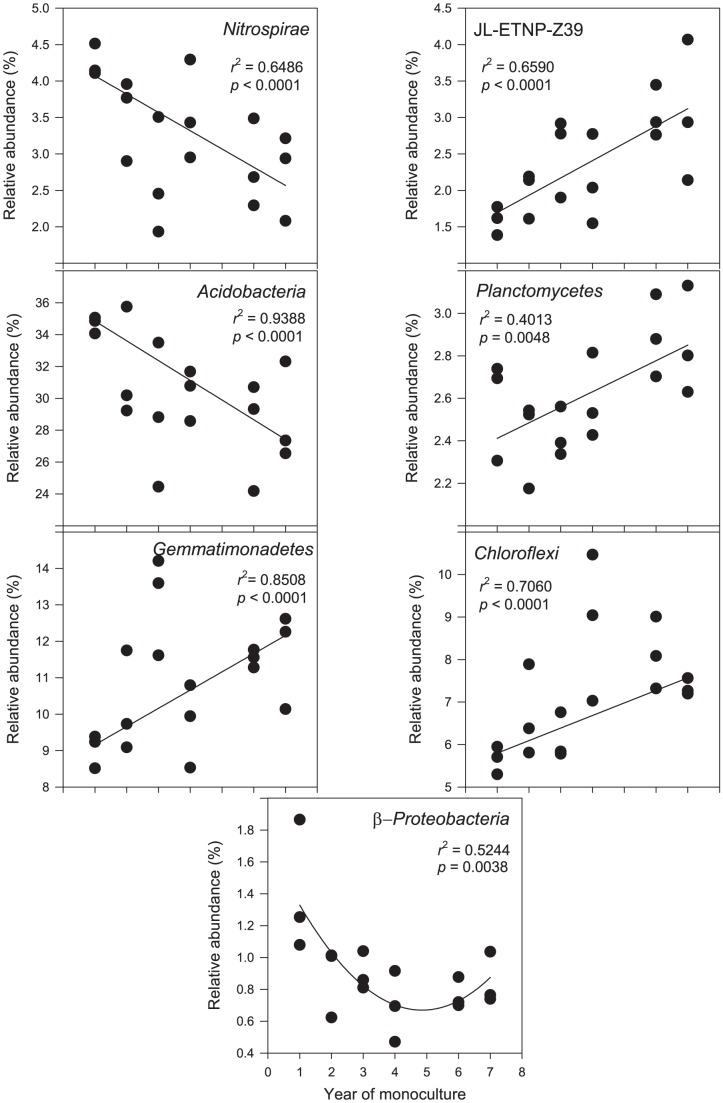
Regressions between bacterial taxa abundances and the year of potato monoculture.

To determine what bacterial taxa were significantly changed over time (the year of monoculture), correlations between taxa abundances and time were conducted. In total, seven taxa showed significant correlations (Table 1). The sum of average relative abundances of these taxa was 57.6%. *Acidobacteria* and *Nitrospirae* were linearly decreased in abundance (Figure 4), whereas *Gemmatimonadetes*, *Chloroflexi*, *Planctomycetes*, and JL-ETNP-Z39 were linearly increased. β-*Proteobacteria* was also decreased but was best fitted with a non-linear model.

**Table 1:** Correlation coefficient between abundance of microbial taxa and the year of monoculture^a^.

Taxa	Average RA (%)	Pearson	Kendall *τ_b_*	Spearman *ρ*
*Acidobacteria*	30.4	−0.550*	−0.424*	−0.561*
*Gemmatimonadetes*	10.9		0.369*	0.486*
*Chloroflexi*	7.1	0.524*	0.466**	0.649**
*Nitrospirae*	3.3	−0.554*	−0.452*	−0.561*
*Planctomycetes*	2.6	0.618**	0.424*	0.567*
JL-ETNP-Z39	2.4	0.706**	0.522**	0.680**
β*-Proteobacteria*	0.9	−0.500*	−0.369*	−0.505*

a RA is relative abundances. * and ** represent significance level at <0.05 and <0.01 respectively. The taxa with average relative abundance over 18 samples <0.5% and with significance level >0.05 were not shown.

### Regression between *Fusarium* and other bacterial taxa in abundances

**Figure 5:**
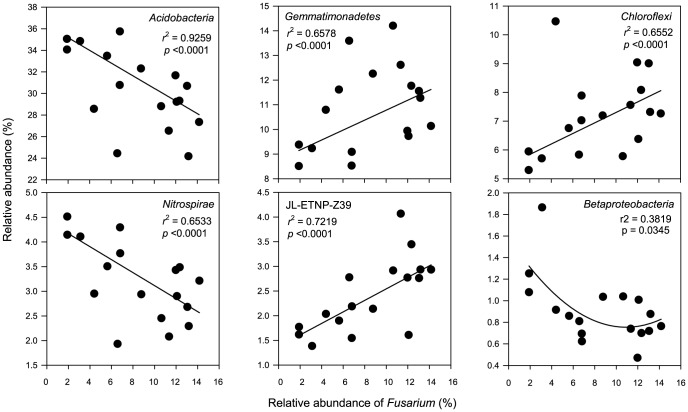
Regressions between relative abundance of *Fusarium* and bacterial taxa. Regressions with *p* values >0.05 are not shown.

Regressions between *Fusarium* and the bacterial taxa abundances showed that among the seven taxa that significantly changed over time (Table 1), six showed significant regressions (Figure 5). *Acidobacteria* and *Nitrospirae* showed negative linear regressions whereas β*-Proteobacteria* showed negative non-linear regression. *Gemmatimonadetes*, *Chloroflexi* and JL-ETNP-Z39 showed positive linear regressions.

## Discussion

The relative abundance rank profiles of fungal and bacterial phyla reported in this study generally agree with previous pyrosequencing surveys of soil microbial communities. As for bacteria, *Acidobacteria* and α-*Proteobacteria* were the top two abundant phyla across all samples (Figure 1), agreeing with the abundance rank profiles derived from 88 soils collected from North and South America [1], as well as from forest and grassland soils in Germany[2]. Acosta-Martínez et al [36] also reported that *Actinobacteria* and *Bacteriodetes* were the top two phyla in Texas (USA) soils with different managements. The average relative abundance of *Gemmatimonadetes* in our samples was 10.9% (Figure 1), which is much higher than the <3% reported in previous studies [1], [2], [36]. DeBruyn et al [37] reported that *Gemmatimonadetes* is a drier soil-adapted phylum, which is probably the reason why its abundance was so high in our soils. As for fungi, *Ascomycota* was the top abundant phylum in our samples, accounting for 58.2% in average relative abundance, generally agreeing with the results reported by Buée et al [38] and by McGuire et al [39] for forest soils. However, our results contrasted with those derieved from tallgrass prairie soils [40], in which basidiomycetes (*Basidiomycota*) accounted for 54% relative abundance and ascomycetes (*Ascomycota*) accounted for 41%. In our samples, *Basidiomycota* accounted for 0.6% only. The difference was probably due to different soils in nature. Different primers targeting different genes/regions and different databases used for phylogentical assignment also affected the results.

Using the soil samples along a year-gradient of potato monoculture, several facts about potato monoculture and soil microbial communities were revealed. First, Pearson, Kendall, and Spearman correlation methods were used simultaneously in order to catch all possible significant correlations between bacterial taxa and the years of monoculture. Different methods have different merits. Pearson method measures the degree of linear dependence between two variables, Kendall measures the similarity of the orderings of the variables when ranked by each of the quantities (rank correlation), and Spearman measures how well the relationship between two variables can be described using a monotonic function. Generally speaking, Pearson measures linear relationships, whereas Kendall and Spearman measure nonlinear relationships. Results showed that the majority of the bacterial community (57.6% by abundance) was significantly changed over time (Table 1), including the top abundant taxon *Acidobacteria*. Bacterial diversity and richness indices were significantly and linearly decreased with an increase of monoculture time (year), but the corresponding values for fungal community was nearly unaffected (Figure 1). This suggests differential responses of bacterial and fungal communities to potato monoculture. Using fatty acid methyl ester profiles to characterize soil microbial communities, several other studies [41], [42], [43], [44], [45] also reported differential responses of fungal and bacterial communities to potato monoculture. Larkin and Honeycutt [42], for example, reported that continuous potato resulted in lower proportions of polyunsaturated fatty acids, which are most closely associated with fungi, resulting in a lower fungi-to-bacteria ratio than several other rotations. By substrate utilization approach, Larkin et al [44] reported that potato monoculture produced the lowest values in soil microbial activity, richness and diversity among other rotation treatments. Although these studies did not use high throughput sequencing approach as present study did, similar conclusion was drawn, suggesting that the differential responses of fungal and bacterial communities were common in potato monoculture agroecosystems. However, whether differential responses exist in other crop monoculture agroecosystems is not clear. A few studies have reported decreased bacterial diversities in monoculture ecosystems of rice [21], cotton [36], soybean [22] and watermelon [46]. Unfortunately, these studies did not include fungal communities, or they were included but such differential responses were not looked for. Nevertheless, our results point out that the link between microbial diversity and soil quality/sustainability depends on the focus of the microbial community, i.e. bacterial or fungal. In the present case, the soil sustainability decline represented by a potato yield decline and disease incidence increase (Figure 2) corresponded to a decrease in bacterial, but not fungal, diversity.

Second, *Fusarium* was the only soilborne pathogen-associated fungal genus substantially detected by our present high-throughput pyrosequencing protocol (Table S1 and Table S2), and was continuously and significantly enriched (Figure 2). The change pattern of *Fusarium* was closely associated with disease incidence and yield decline, suggesting its reponsibility for yield decline. *Fusarium* enrichment in soils is reportedly related to disease resistance of cultivars used [46], [47]. The cultivar “Atlantic” used in the present experiment was reportedly susceptible to *Fusarium roseum* [48]. Root exudates and debris left in soils from previous crops, especially a monoculture potato crop that was sampled in this study, would likely favor *Fusarium* proliferation and enrichment in soils.

Third, *Fusarium* enrichment over time was correlated with soil variables. Among the six selected variables, OM and TN were significantly and negatively correlated with *Fusarium* abundances, and EC was significantly and positively correlated (Figure 3). These three correlated soil variables are all associated with field management. The site of the experimental plots for this study was located in an arid region where the mean annual precipitation is only 186 mm. Cations introduced from fertilizers are not easily leached out of the soils, leading to an increase of EC values (Figure S2). Arid soils are also water limited. Irrigation improves the soil water regime, promotes microbial activity, thus enhancing decomposition of soil OM [49]. Without organic amendments to compensate OM loss (as in the present experiment), soil OM content will be inevitably decreased (Figure S2), instead of being preserved by dryness. TN will be decreased as well because it is mostly associated with soil OM. Therefore, soil management causes change in the soil variables of OM, TN and EC, thus causing *Fusarium* enrichment in soils.

Fourth, among the seven taxa that were significantly changed over time under continued potato monoculture pressure (Table 1), *Acidobacteria* and *Nitrospirae* were linearly decreased over time in abundance (Figure 4). This is probably relevant to their ecophysiologies. As for *Acidobacteria*, it has been shown that the abundance of *Acidobacteria* was negative correlated with soil carbon mineralization rates [50]. Although the carbon mineralization rate was not measured in this study, it was reasonable to believe that microbial activity and carbon mineralization rates in the arid soils were increased due to the improved water regime by irrigation, which consequently led to the decrease of soil OM contents (Figure S2). Therefore our data indirectly supported the ecological classification of *Acidobacteria* as an oligotrophic phylum. As for *Nitrospirae*, its ecophysiology is not clear. However, there have been evidences indicating that it tended to adapt to substrate-limited and well-aerated conditions [51], [52], implying that the ecophysiology of *Nitrospirae* was similar to that of *Acidobacteria*. Furthermore, *Nitrospirae* is a phylum most likely involved in nitrite oxidation [51], [52], although phylogenetic heterogeneity implies it has diverse functions. Nevertheless, the decrease of *Nitrospirae* abundance suggested negative effects on the process of soil nitrification.

Fifth, six bacterial taxa were significantly correlated with the abundance of *Fusarium* (Figure 5). Positive correlations were found between *Fusarium* and *Gemmatimonadetes*, *Chloroflexi* and JL-ETNP-Z39. Negative correlations occurred between *Fusarium* and *Acidobacteria*, *Nitrospirae* and β*-Proteobacteria*. The sum of average relative abundances of these correlated bacterial taxa was 55% (Figure 5), suggesting that potato monoculture not only enriched *Fusarium* but also shaped the overall soil bacterial communities by enriching and impoverishing certain bacterial taxa. Furthermore, the data also suggested these taxa exhibited similar behaviors in responses to potato monoculture and/or soil variables, providing additional insights into the ecological behaviors of different taxa in the environments.

## Conclusion

Fungal and bacterial communities exhibited different responses to potato monoculture. Fungal diversity was nearly unaffected, whereas bacterial diversity was significantly decreased over time (years). Seven bacterial taxa were significantly changed, including the top abundant taxa *Acidobacteria* and *Gemmatimonadetes*. *Fusarium* was the only soilborne pathogen-associated fungal genus substantially detected by our high throughput pyrosequencing approach. *Fusarium* became enriched over the experimental time span, and was closely associated with potato yield decline and disease increases. *Fusarium* abundance was also significantly and negatively correlated with soil OM and TN, but significantly and positively with EC. *Fusarium* was positively correlated with abundances of *Gemmatimonadetes*, *Chloroflexi*, and JL-ETNP-Z39, and negatively correlated with *Acidobacteria*, *Nitrospirae*, and β*-Proteobacteria* abundances. These correlations suggested their similar behaviors in responses to potato monoculture and/or soil variables, providing further insights into the effects of potato monoculture on soil microbial communities.

## Supporting Information

Figure S1 **The changing trends in abundances of fungal taxa over time.** Phylogenetic lineages were assigned by order or higher levels.
(PDF)

Figure S2 **Regression between soil variable and the year of monoculture.**
(PDF)

Table S1 **Relative abundance of genus within the order Hypocreales and Sodariales.**
(XLSX)

Table S2 **The relative abundance of pathogen-associated fungal genus detected in the soil samples.**
(DOCX)
