# Supplementary figures and images for: Microbial Community Diversities and Taxa Abundances in Soils along a Seven-Year Gradient of Potato Monoculture Using High Throughput Pyrosequencing Approach
Source: PLoS One. 2014 Jan 30;9(1):e86610. doi: 10.1371/journal.pone.0086610 (PMC3907449; doi:10.1371/journal.pone.0086610)

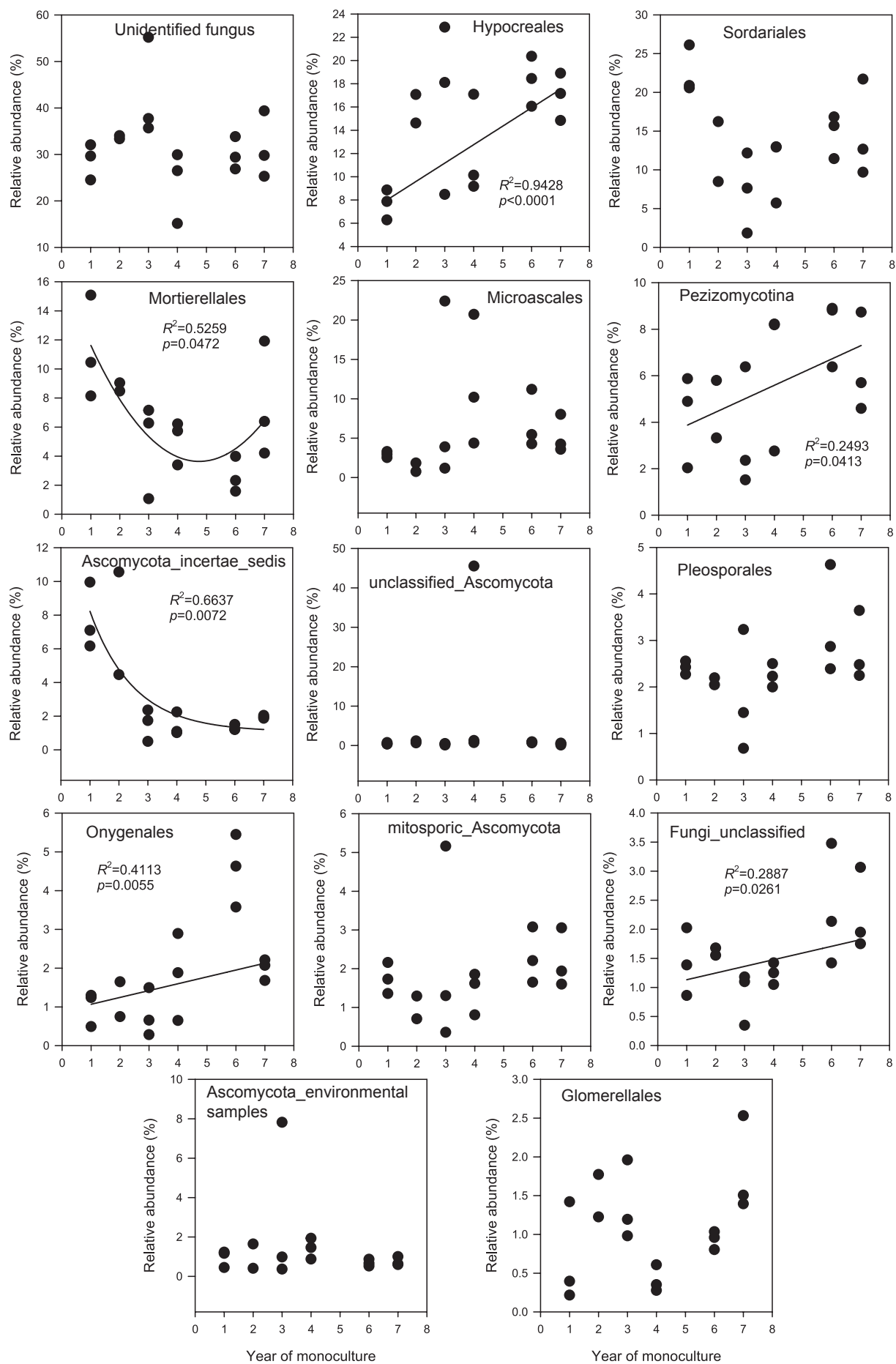

Supplement: Figure S1 — The changing trends in abundances of fungal taxa over time. Phylogenetic lineages were assigned by order or higher levels. (PDF) [file pone.0086610.s001.pdf]

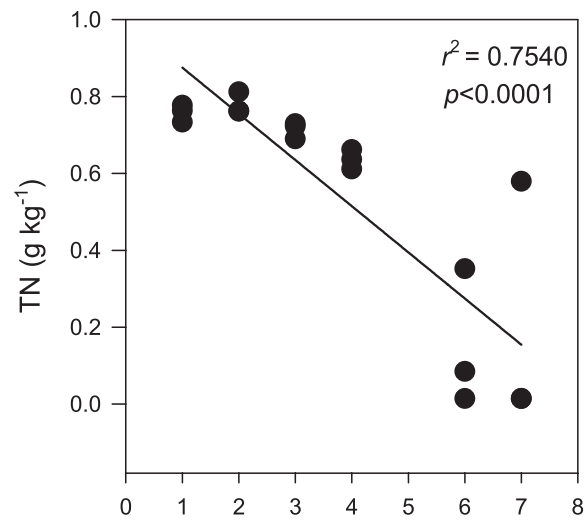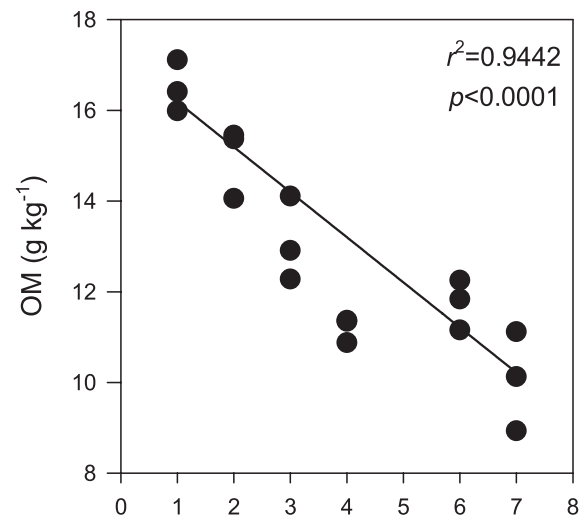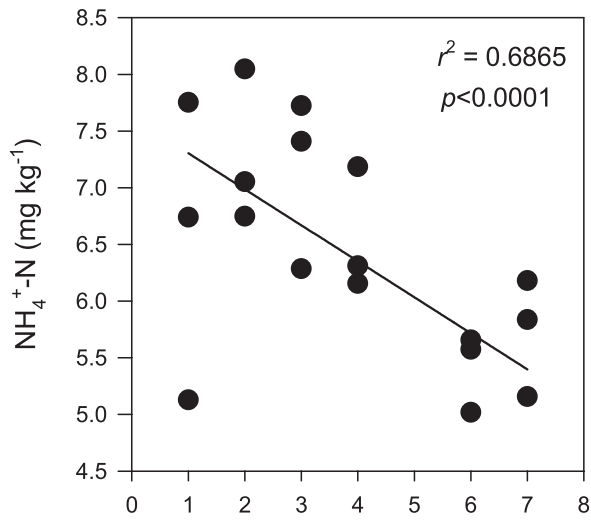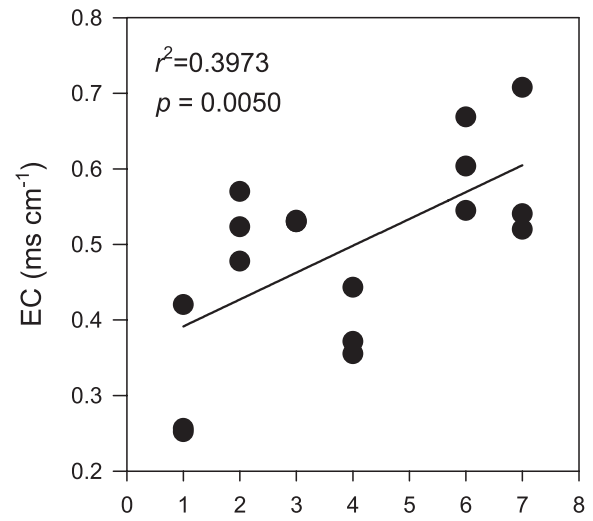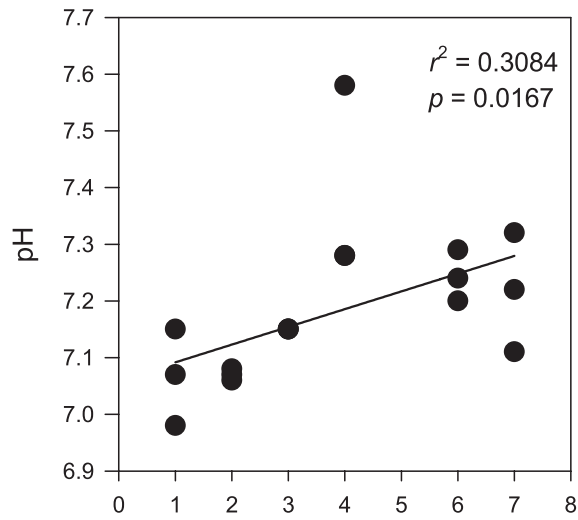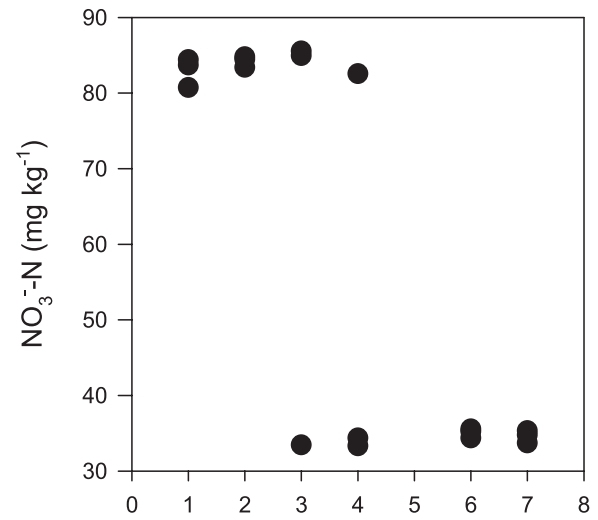

Year of monoculture

Year of monoculture

Supplement: Figure S2 — Regression between soil variable and the year of monoculture. (PDF) [file pone.0086610.s002.pdf]
