# Supplementary material for: Microbial Community Diversities and Taxa Abundances in Soils along a Seven-Year Gradient of Potato Monoculture Using High Throughput Pyrosequencing Approach
Source: PLoS One. 2014 Jan 30;9(1):e86610. doi: 10.1371/journal.pone.0086610 (PMC3907449; doi:10.1371/journal.pone.0086610)
Supplement: Table S2 — The relative abundance of pathogen-associated fungal genus detected in the soil samples. (DOCX) [file pone.0086610.s004.docx]

**Table S2. The relative abundance of pathogen-associated fungal genus detected in the soil samples.**

| Known potato fungal disease ^a^ | Known responsible fungal pathogen ^a^ | RA (%) ^b^ |
| --- | --- | --- |
| Black dot | *Colletotrichum coccodes* | <0.02 |
| Fusarium dry rots | *Fusarium* sps*.* | 1.89-14.11 |
| Silver scurf | *Helminthosporium solani* |  |
| Charcoal rot | *Macrophomina phaseolina* |  |
| Phoma leaf spot/ Gangrene | *Phoma* sps*.* | <0.97 |
| Pink rot | *Phytophthora erythroseptica* |  |
| Skin spot | *Polyscytalum pustulans* |  |
| Leak | *Pythium ultimum* var*. ultimum* | <0.01 |
| Black scurf/Stem canker | *Rhizoctonia solani* |  |
| Rosellinia black rot | *Rosellinia* sps*.* |  |
| White mold | *Sclerotinia sclerotinium* |  |
| Stem rot | *Sclerotium rolfsii* |  |
| Powdery scab | *Spongospora subterranea* |  |
| Wart | *Synchytrium endobioticum* | <0.01 |
| Thecaphora smut | *Thecaphora solani* |  |
| Verticillium wilt | *Verticillium dahliae* and *V. albo-atrum* | <0.60 |
| Early blight/ Brown spot and Black pit | *Alternaria* sps*.* | <0.51 |

^a^ Known potato fungal diseases and responsible soilborne fungal pathogens are listed according to literatures [34,35]. *Alternaria* sps*.* are not recognized soilborne pathogens but also listed.

^b^ RA stands for relative abundance, and is the genus-based sum detected in present soil samples.
